# Screening potential biomarkers associated with insulin resistance in high-fat diet-fed mice by integrating metagenomics and untargeted metabolomics

**DOI:** 10.1128/spectrum.04094-23

**Published:** 2024-02-27

**Authors:** Yunyan Zhou, Jiahui Tang, Wei Du, Yong Zhang, Bang-Ce Ye

**Affiliations:** 1 Institute of Engineering Biology and Health, Collaborative Innovation Center of Yangtze River Delta Region Green Pharmaceuticals, College of Pharmaceutical Sciences, Zhejiang University of Technology, Hangzhou, China; 2 Laboratory of Biosystem and Microanalysis, State Key Laboratory of Bioreactor Engineering, East China University of Science and Technology, Shanghai, China; Huazhong University of Science and Technology, Wuhan, China

**Keywords:** insulin resistance, gut microbiota, metagenomics, non-targeted metabolomics, biomarkers

## Abstract

Insulin resistance is the primary pathophysiological basis for metabolic syndrome and type 2 diabetes. Gut microbiota and microbiota-derived metabolites are pivotal in insulin resistance. However, identifying the specific microbes and key metabolites with causal roles is a challenging task, and the underlying mechanisms require further exploration. Here, we successfully constructed a model of insulin resistance in mice induced by a high-fat diet (HFD) and screened potential biomarkers associated with insulin resistance by integrating metagenomics and untargeted metabolomics. Our findings showed a significant increase in the abundance of 30 species of *Alistipes* in HFD mice compared to normal diet (ND) mice, while the abundance of *Desulfovibrio* and *Candidatus Amulumruptor* was significantly lower in HFD mice than in ND mice. Non-targeted metabolomics analysis identified 21 insulin resistance-associated metabolites, originating from the microbiota or co-metabolized by both the microbiota and the host. These metabolites were primarily enriched in aromatic amino acid metabolism (tryptophan metabolism, tyrosine metabolism, and phenylalanine metabolism) and arginine biosynthesis. Further analysis revealed a significant association between the three distinct genera and 21 differentiated metabolites in the HFD and ND mice. Kyoto Encyclopedia of Genes and Genomes (KEGG) analysis of representative genomes from 12 species of the three distinct genera further revealed the functional potential in aromatic amino acid metabolism and arginine biosynthesis. This study lays the groundwork for future investigations into the mechanisms through which the gut microbiota and its metabolites impact insulin resistance.

**IMPORTANCE:**

In this study, we aim to identify the microbes and metabolites linked to insulin resistance, some of which have not been previously reported in insulin resistance-related studies. This adds a complementary dimension to existing research. Furthermore, we establish a correlation between alterations in the gut microbiota and metabolite levels. These findings serve as a foundation for identifying the causal bacterial species and metabolites. They also offer insights that guide further exploration into the mechanisms through which these factors influence host insulin resistance.

## INTRODUCTION

Insulin resistance serves as the foundational pathophysiological basis for both metabolic syndrome and type 2 diabetes (1, 2). Numerous studies have elucidated the crucial role of gut microbiota in the development of insulin resistance (3, 4). The administration of a high-fat diet (HFD) has been linked to the dysfunction of mesenteric lymphatic vessels, subsequently fostering abdominal fat deposition and insulin resistance (5). Simultaneously, the administration of HFD induces alterations in the composition of the gut microbiota and gut environment. These changes encompass a reduction in gut bacterial diversity, a shift in the Firmicutes-to-Bacteroides ratio, and the inhibition of the growth of certain beneficial and symbiotic bacteria, coupled with the promotion of the potentially harmful ones (6–8).

Alterations in the composition of the gut microbiota in the host can lead to changes in the profile of gut microbial products. Various classes of microbiota-derived metabolites, especially bile acids, short-chain fatty acids (SCFAs), branched-chain amino acids, trimethylamine*-N*-oxide, tryptophan, and indole derivatives, have been shown to be implicated in the pathogenesis of metabolic disorders, including insulin resistance (9). Nevertheless, the composition of the gut microbiota is influenced by various factors, including genetics, environment, diet, and physiology. These differences contribute to variations in host metabolism (10). Hence, identifying the specific microbes and key metabolites with causal roles poses a challenging and intricate task, demanding further exploration of the underlying mechanisms.

Metagenomics provides insights into the composition and abundance of microbial communities and functional genes. Metabolomics involves the qualitative and relative quantitative analyses of small-molecule metabolites in biological samples, employing liquid chromatography-tandem mass spectrometry (LC-MS/MS). Integrating microbiome and metabolome analyses can establish a logical relationship between microbe and metabolite phenotype (11, 12). In this study, we screened potential gut microbiota and metabolite biomarkers associated with insulin resistance in mice induced by an HFD, integrating metagenomics and untargeted metabolomics. Additionally, we analyzed the association between differential microbiota and metabolites, revealing potential relationships between microbes and metabolites, and insulin resistance.

## RESULTS

### Construction of an insulin resistance mouse model

The 5-week-old male C57BL/6J mice were fed HFD to construct an insulin resistance mouse model. Over the 15 weeks of HFD feeding, mice fed the high-fat diet exhibited noteworthy weight gain and elevated levels of serum fasting blood glucose (Fig. S1A and B). After 15 weeks of HFD feeding, the homeostasis model assessment of insulin resistance (HOMA-IR) for mice on the high-fat diet was significantly higher compared to those on the normal diet (ND) (Fig. S1C). These results indicated the successful construction of the insulin resistance mouse model.

### Identification of potential bacterial taxa showing differential abundances between ND and HFD mice

**Fig 1:**
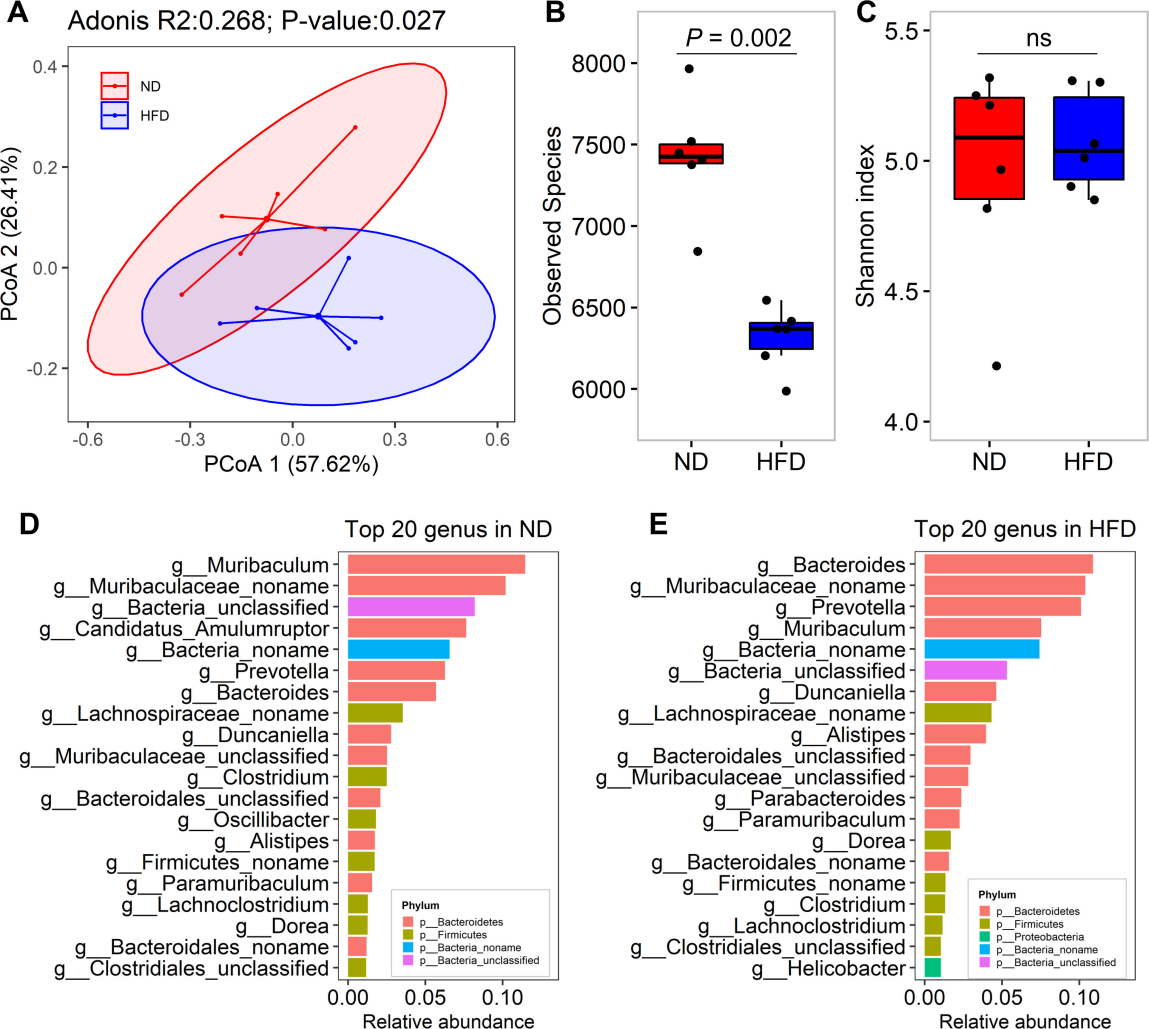
Comparison of gut microbiota composition between ND and HFD mice. (**A**) PCoA of gut microbiota in ND and HFD mice. PCoA, based on the Bray–Curtis distance matrix at the species level, demonstrates the clustering of samples. The comparison of the *β*-diversity of the gut microbiome between ND and HFD mice was performed using Adonis analysis, and *P* < 0.05 was set as the significance threshold. (**B**) Comparison of the richness (number) of species between ND and HFD mice. Six animals were used for each group. (**C**) Comparison of the evenness (Shannon index) of species between ND and HFD mice. The comparisons were performed using the Wilcoxon rank sum test, and a *P*-value <0.05 corrected for multiple tests (false discovery rate) was set as the significance threshold. (**D**) The top 20 highly abundant genus enriched in the ND mice. (**E**) The top 20 highly abundant genus enriched in the HFD mice. Different colors represent different phyla.

Bacteroidetes (46.4% ± 15.2%) and Firmicutes (14.9% ± 7.1%) dominated the gut microbiota, while a considerable proportion (bacteria unclassified: 32.7 %± 6.6%) remained unclassified at the phylum level (Fig. S2A). The comparative analysis showed that the ratio of Firmicutes to Bacteroidetes did not differ significantly between HFD and ND mice (Fig. S2B, *P* > 0.05). At the species level, principal coordinates analysis (PCoA) revealed noticeable differences in the gut microbiota structure between ND and HFD mice (Fig. 1A, *P* = 0.027). The richness (the number of observed species) in gut microbiota communities significantly decreased in HFD-fed mice (Fig. 1B, *P* = 0.002). However, no significant difference was observed in the evenness (Shannon index) between the ND and HFD groups (Fig. 1C, *P* > 0.05).

Eighteen bacterial genera were common among the top 20 dominant genera in terms of relative abundance in both the ND and HFD groups. These included *Bacteroides*, *Prevotella*, *Muribaculum*, *Duncaniella*, *Alistipes*, and *Paramuribaculum* belonging to the phylum Bacteroidetes, as well as *Dorea*, *Clostridium*, and *Lachnoclostridium* belonging to the phylum Firmicutes. However, the relative abundances of these dominant bacterial genera and their ranking among all bacteria were altered in the gut microbiota of HFD mice (Fig. 1D and E).

**Fig 2:**
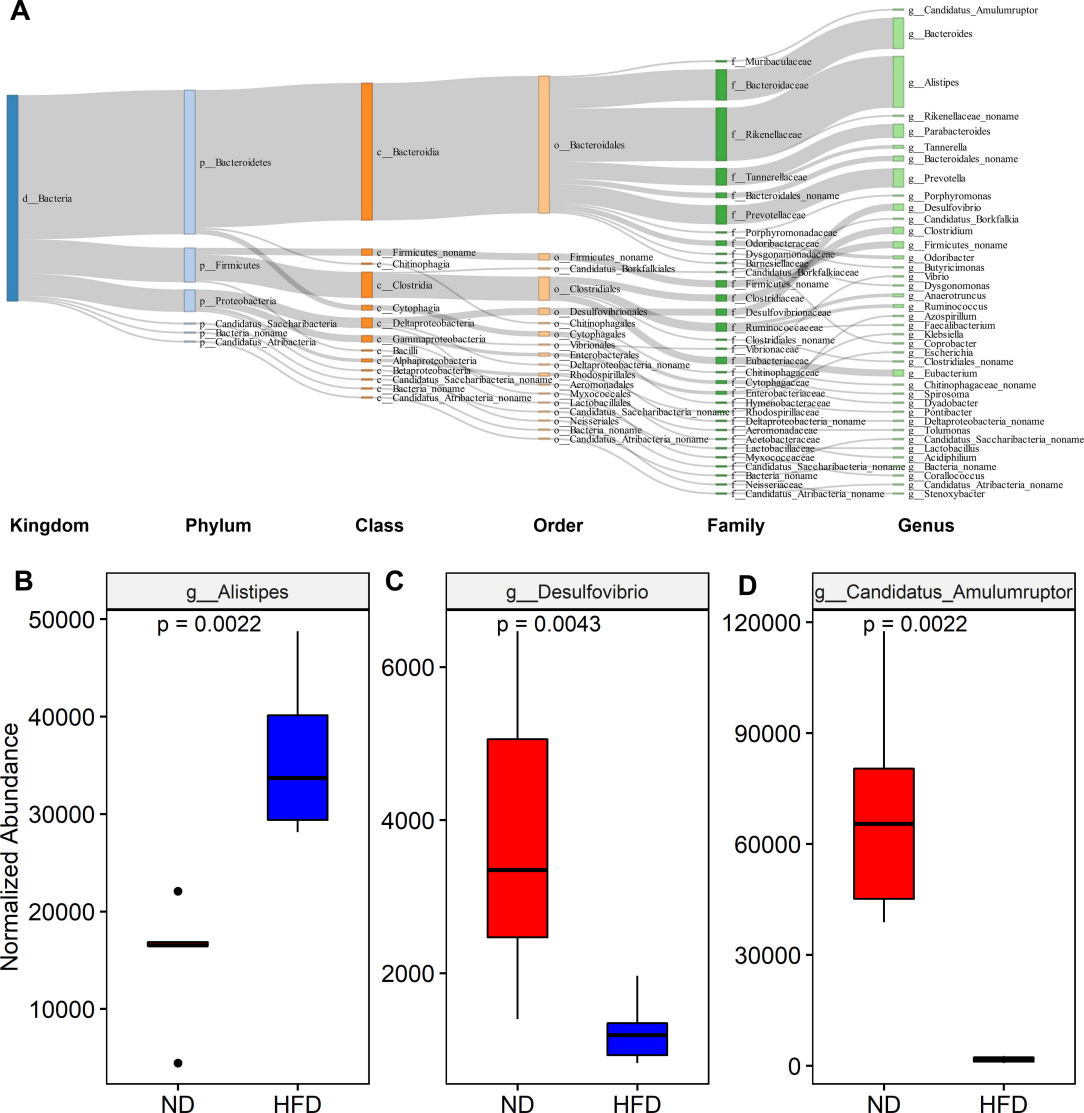
The taxa showing significantly differential abundance between ND and HFD mice. (**A**) Taxonomic distribution of 120 species showing significantly differential abundance between ND and HFD groups at various taxonomy levels. The colors of the rectangles represent different taxonomy levels. The length of the rectangles indicates the number of taxa. (**B–D**) Significant difference in the abundance of *Alistipes*, *Desulfovibrio*, and *Candidatus Amulumruptor* between ND and HFD groups. Six animals were used for each group. The comparisons were performed using the Wilcoxon rank sum test, and a *P*-value <0.05 corrected for multiple tests (false discovery rate) was set as the significance threshold.

To further investigate the specific changes in the gut microbiota communities, the abundance of the predominant species was compared between the ND and HFD groups. A total of 924 species with a relative abundance >0.01% were used for comparative analysis between groups. These species accounted for 97% of the total abundance of all bacterial species. A total of 120 species showed differential abundance between the two groups (Table S1). The *Sankey* diagram illustrates the distribution of these 120 species at different taxonomy levels (Fig. 2A). Among these, 74 significantly different species were enriched in HFD mice, including 30 species from *Alistipes*, 17 species from *Bacteroides*, and 7 species from *Parabacteroides* (Table S1). Among the top 20 highly abundant species enriched in the HFD mice, there were 12 species from *Alistipes*, 4 species from *Bacteroides*, and 1 species from *Parabacteroides* (Table S2). Conversely, 46 species were significantly lower in HFD mice than in ND mice, including 9 species from *Prevotella*, 4 species from *Desulfovibrio*, 4 species from *Eubacterium*, and 3 species from *Clostridium* (Table S1). In the top 20 highly abundant species enriched in ND mice, there were one species from *Prevotella*, four species from *Desulfovibrio*, and three species from *Clostridium* (Table S2). Among these bacterial genera containing multiple different species, *Alistipes* (*P* = 0.0022) and *Desulfovibrio* (*P* = 0.0043) have significantly different abundance in the ND and HFD groups (Fig. 2B and C). In addition, *Candidatus Amulumruptor*, one of the top 20 abundant genera in the ND group, also had a significantly different abundance between the ND and HFD groups (Fig. 2D, *P* = 0.0022), although *C. Amulumruptor caecigallinarius* with a significantly different abundance between the two groups was not one of the top 20 abundance species in the ND group (Table S2). These results suggest a high correlation between the occurrence and aggravation of insulin resistance and the dynamic abundance changes of specific bacterial species.

### KEGG pathways show differential enrichments between ND and HFD mice

**Fig 3:**
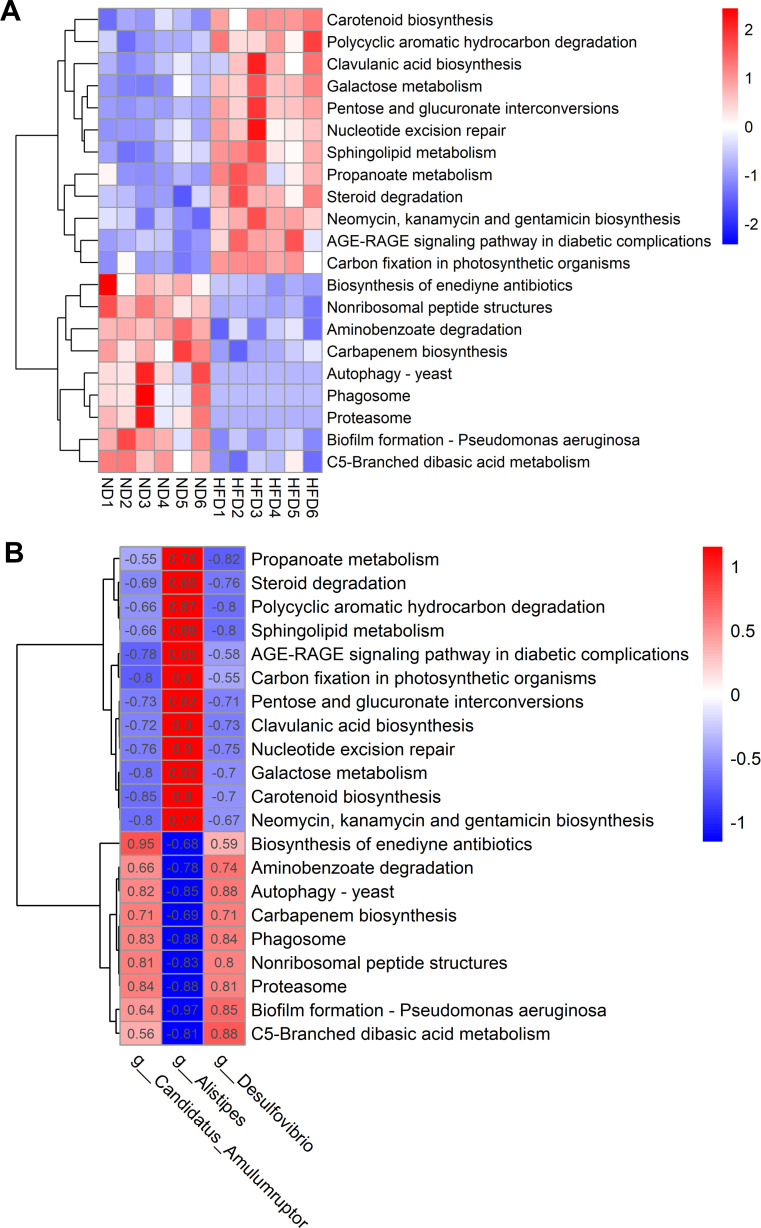
Significantly different metabolic pathways between ND and HFD mice. (**A**) Twenty-one KEGG pathways showing differential enrichments between ND and HFD mice. (**B**) Correlation between the 21 KEGG pathways and the three key genera *Alistipes*, *Desulfovibrio*, and *Candidatus Amulumruptor*.

A total of 21 KEGG pathways exhibited distinct enrichments between ND and HFD mice (*P* < 0.05). Of these, 12 pathways were enriched in the HFD group, encompassing sphingolipid metabolism, propanoate metabolism, and advanced glycation end products - receptor for advanced glycation end products (AGE-RAGE) signaling pathway in diabetic complications (Fig. 3A). Conversely, nine pathways exhibited enrichment in the ND group, involving aminobenzoate degradation, biosynthesis of enediyne antibiotics, and carbapenem biosynthesis (Fig. 3A). Furthermore, these 21 KEGG pathways demonstrated a significant correlation with *Alistipes*, *Desulfovibrio*, and *C. Amulumruptor*, three potential bacterial genera associated with insulin resistance (Fig. 3B). Implying that alterations in these metabolic pathways may be linked to variations in the abundance of these three genera within the gut microbiome.

### Differences in the fecal metabolome between ND and HFD mice

**Fig 4:**
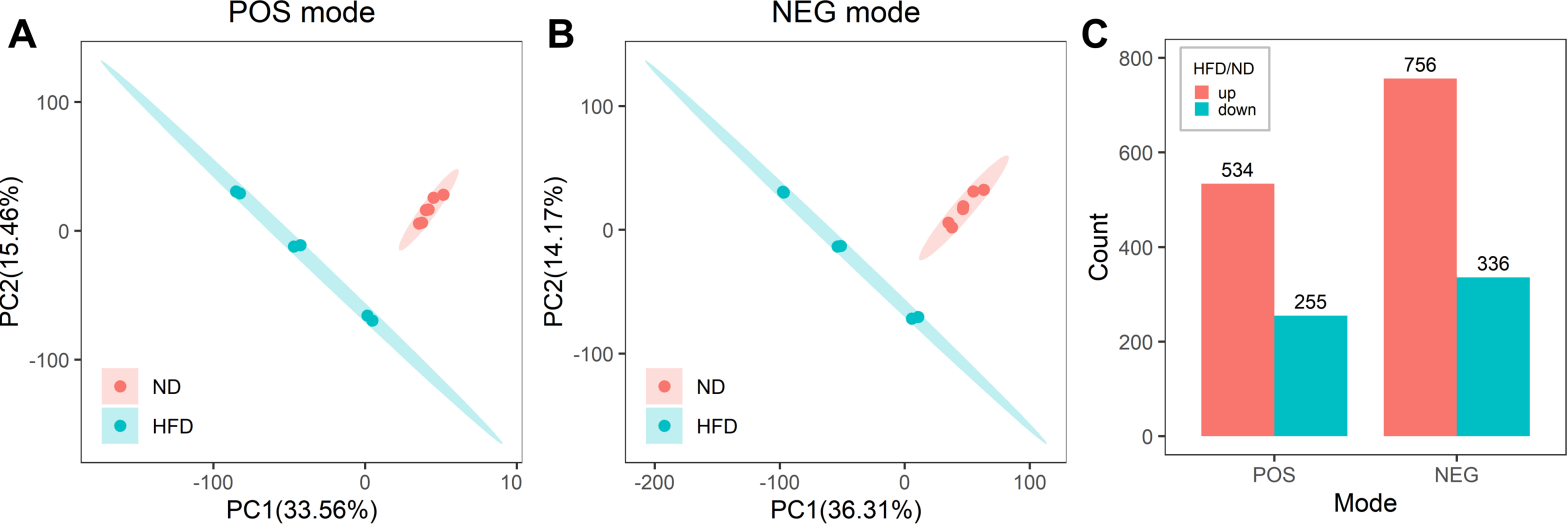
Differences in the fecal metabolome between ND and HFD mice. (**A**) Differences in the composition of metabolites between ND and HFD groups in positive (POS) ion mode. (**B**) Differences in the composition of metabolites between ND and HFD groups in negative (NEG) ion mode. (**C**) Number of metabolites enriched in ND and HFD mice in POS and NEG modes. Six animals were used for each group.

A total of 7,813 and 9,633 high-quality metabolites were used for differential analysis in the POS and NEG models, respectively. Principal Component Analysis (PCA) showed that the composition of metabolites was significantly different between the ND and HFD groups (Fig. 4A and B). In the POS mode, there were 534 metabolites significantly upregulated and 255 metabolites significantly downregulated in the HFD group (Fig. 4C). In the NEG mode, 756 metabolites were significantly upregulated and 336 metabolites were significantly downregulated in the HFD group (Fig. 4C). These findings collectively indicate a significant alteration in the fecal metabolome of HFD-induced insulin-resistant mice compared to ND mice.

**Fig 5:**
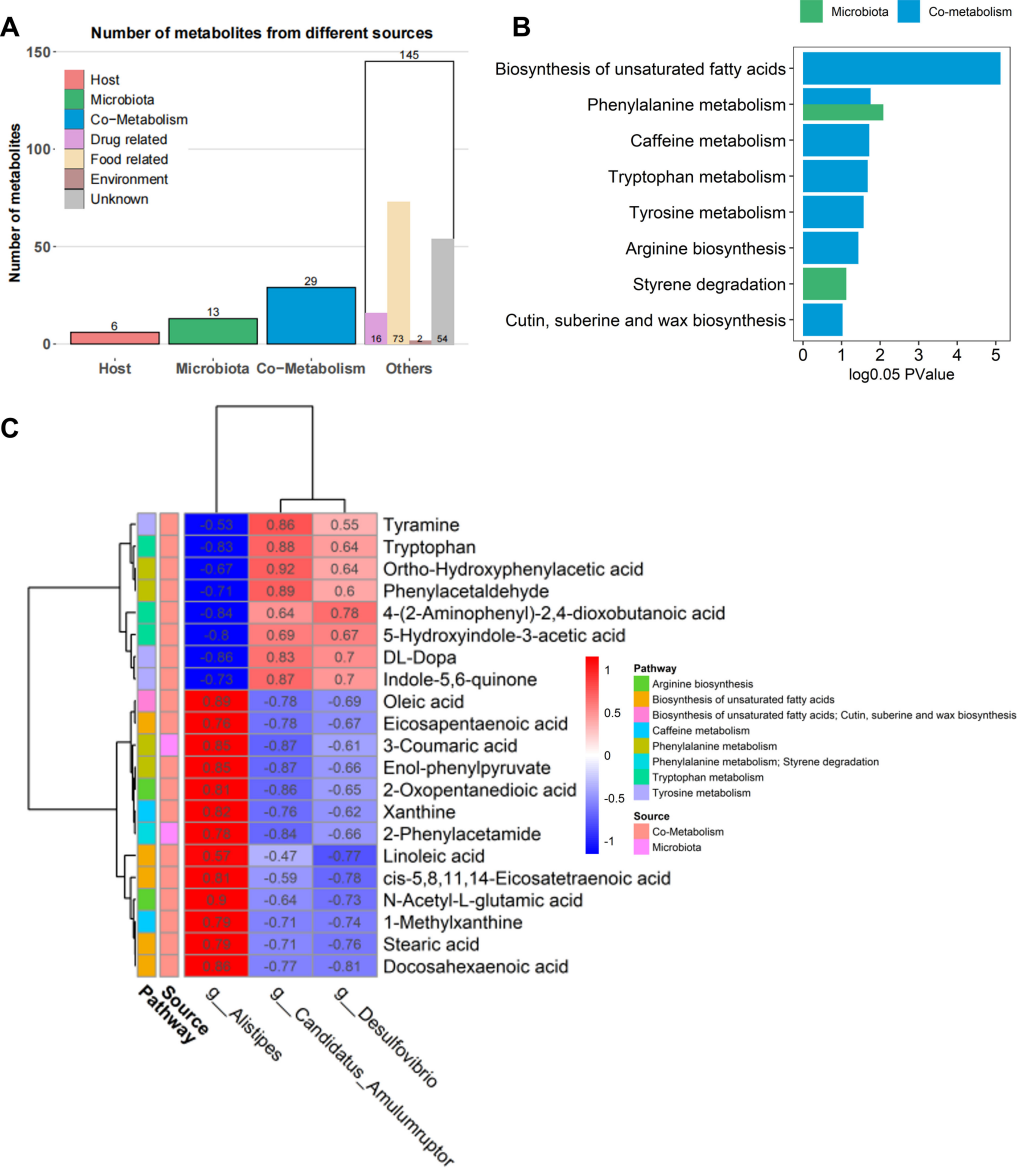
The sources and enriched metabolic pathways of different metabolites in ND and HFD groups. (**A**) Number of metabolites of different sources. (**B**) Metabolic pathways enriched by the 43 microbiota or cometabolism metabolites with significantly different abundances between ND and HFD groups. (**C**) Correlation between 21 metabolites and 3 key genera *Alistipes*, *Desulfovibrio*, and *Candidatus Amulumruptor*. Six animals were used for each group.

To streamline subsequent analysis, we merged the metabolites of the POS and NEG modes, exhibiting significant differences between the ND and HFD groups. The focus was on known metabolites at the level 2 fragment ion in the database. In cases where the metabolite name was duplicated, the selection prioritized those with smaller coefficient of variation values. Ultimately, 194 metabolites were employed for subsequent analyses, including the identification of potential metabolite biomarkers associated with insulin resistance, source analysis of metabolites, and enrichment analysis of metabolic pathways. Given our interest in the role of gut microbiota in insulin resistance, the initial analysis focused on categorizing the source of 194 metabolites, i.e., whether they originate from the host, microbiota, or both. Results revealed that 13 metabolites were derived from the microbiota and 29 metabolites were cometabolisms (co-produced by both host and microbiota). Most of the 194 metabolites (46%) were food- or drug-related metabolites, and the origin of 28% of the metabolites was unknown (Fig. 5A). The 43 microbiota or cometabolism metabolites were enriched in 43 metabolic pathways. According to the different origins of the metabolites, phenylalanine metabolism and styrene degradation were metabolic pathways shared by both the microbiota and cometabolism (Table S3). We then performed an enrichment analysis of metabolic pathways according to the differential metabolites from different origins. Eight metabolic pathways, covering 21 significantly different metabolites, showed significant differences between the ND and HFD groups (log0.05 *P*-value > 1), including tryptophan metabolism, tyrosine metabolism, phenylalanine metabolism, and arginine biosynthesis (Fig. 5B, Table S3). Notably, the biosynthesis of unsaturated fatty acids emerged as the most significantly different metabolic pathway between the ND and HFD groups (Fig. 5B).

### Correlation between the changes in the gut microbiome and the shifts in the fecal metabolome

To explore the potential relationship between alterations in the fecal metabolome induced by a high-fat diet and shifts in gut microbiota, we focused on the 3 key differential genera and 21 metabolites involved in eight differential metabolic pathways between the ND and HFD groups. Of the 21 metabolites, 11 were associated with the metabolic pathway of aromatic amino acids, i.e., tryptophan metabolism, tyrosine metabolism, and phenylalanine metabolism. The results indicated that six metabolites in the biosynthesis of unsaturated fatty acids, including oleic acid, eicosapentaenoic acid, linoleic acid, *cis*-5,8,11,14-eicosatetraenoic acid, stearic acid, and docosahexaenoic acid, showed a significant positive correlation with *Alistipes* (Fig. 5C). Metabolites in tryptophan metabolism and tyrosine metabolism, comprising tryptophan, 5-hydroxyindole-3-acetic acid, 4-(2-aminophenyl)-2,4-dioxobutanoic acid, dl-Dopa, tyramine, and indole-5,6-quinone, exhibited a positive correlation with *C. Amulumruptor* and *Desulfovibrio* (Fig. 5C). Notably, 3-coumaric acid, 2-phenylacetamide, and enol-phenylpyruvate, three metabolites involved in the pathway of phenylalanine metabolism, demonstrated a positive correlation with *Alistipes*, the genus enriched in HFD mice. However, ortho-hydroxyphenylacetic acid and phenylacetaldehyde, the two metabolites also from the pathway of phenylalanine metabolism, showed a positive correlation with *C. Amulumruptor* and *Desulfovibrio*, the genera enriched in ND mice (Fig. 5C). Additionally, *Alistipes* demonstrated a significant positive correlation with 2-oxopentanedioic acid and N-Acetyl-L-glutamic acid in the pathway of arginine biosynthesis (Fig. 5C). These results suggest that the changes in the fecal metabolome induced by a high-fat diet are related to changes in the gut microbiota.

### Metabolic potential analysis of three key differential genera

**Fig 6:**
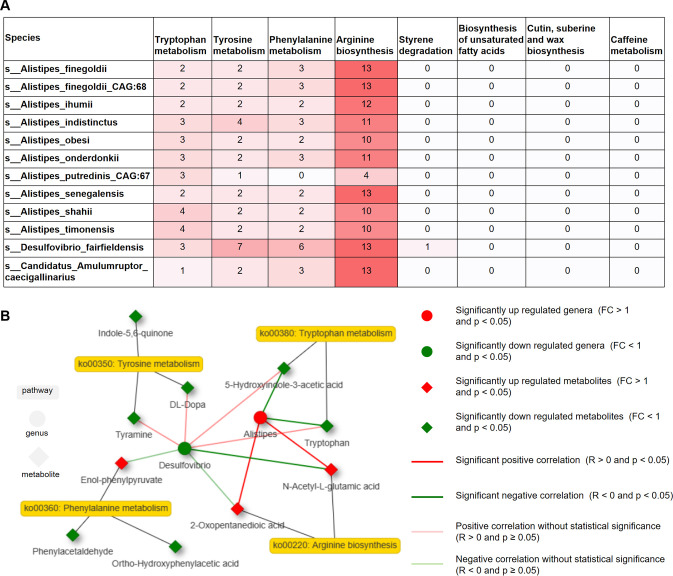
The metabolic potential of *Alistipes*, *Desulfovibrio*, and *Candidatus Amulumruptor*. (**A**) Number of KOs associated with eight important metabolic pathways in the genomes of 12 species from *Alistipes*, *Desulfovibrio*, and *C. Amulumruptor*. The darker the grid background color, the greater the number of KOs associated with this signaling pathway in the genome of the species. (**B**) Interaction network of four key metabolic pathways of tryptophan metabolism, tyrosine metabolism, phenylalanine metabolism, and arginine biosynthesis with their related microbes and metabolites. The red nodes in the network represent significantly upregulated genera or metabolites in the HFD group (FC >1 and *P* < 0.05), and the green nodes in the network represent significantly downregulated genera or metabolites in the HFD group (FC <1 and *P* < 0.05). The red lines between genera and metabolites represent a positive correlation, and the green lines between genera and metabolites represent a negative correlation, and dark red or green represent statistical significance (Spearman rank correlation analyses, *P* < 0.05). Six animals were used for each group.

In the preceding analysis, we identified eight metabolic pathways that significantly differed between the ND and HFD groups and exhibited significant correlations with *Alistipes*, *C. Amulumruptor*, and *Desulfovibrio*, the three key differential genera between the ND and HFD groups (Fig. 5B and C). To further determine the metabolic potential of these three genera, we downloaded the representative genomes of 12 species belonging to these three genera from the National Center for Biotechnology Information (NCBI) RefSeq database and analyzed their potential metabolic capacity by KEGG analysis. These 12 species included *C. Amulumruptor caecigallinarius*, *Desulfovibrio fairfieldensis*, and 10 species with a significantly different abundance between the ND and HFD groups belonging to *Alistipes*. Detailed analyses of KEGG orthology (KO) revealed that KOs associated with tryptophan metabolism, tyrosine metabolism, phenylalanine metabolism, and arginine biosynthesis were present in almost all 12 species (Fig. 6A), implying the functional potential of these species in aromatic amino acid metabolism and arginine biosynthesis. Notably, arginine biosynthesis showed the presence of more than 10 KOs in these species (Fig. 6A). However, these species exhibited almost no KOs related to the styrene degradation, biosynthesis of unsaturated fatty acids, caffeine metabolism, and cutin, suberine, and wax biosynthesis pathways (Fig. 6A).

Subsequently, we constructed an interaction network involving microbes, metabolites, and metabolic pathways. *Alistipes* exhibited a significantly positive correlation with 2-oxopentanedioic acid and N-Acetyl-L-glutamic acid (Spearman correlation, *P* < 0.05), further indicating the functional potential of *Alistipes* in arginine biosynthesis. *Alistipes* was significantly negatively correlated with 5-hydroxyindole-3-acetic acid and tryptophan in the tryptophan metabolism (Fig. 6B). However, 5-hydroxyindole-3-acetic acid and tryptophan showed a positive correlation with *Desulfovibrio*, although not reaching statistical significance. Furthermore, *Desulfovibrio* was significantly negatively correlated with N-Acetyl-L-glutamic acid in arginine biosynthesis (Fig. 6B).

## DISCUSSION

The Firmicutes/Bacteroidetes ratio serves as a marker for gut dysbiosis, particularly associated with conditions such as obesity, type 2 diabetes, and cardiovascular diseases. Our findings indicated that the Firmicutes to Bacteroidetes ratio showed no significant difference between HFD and ND mice. A review highlights that it is currently difficult to associate the Firmicutes/Bacteroidetes ratio with a determined health status due to contradictory results in the literature (13). However, at the species level, we found differences in alpha diversity and the abundance of specific bacterial taxa in gut microbiomes associated with insulin resistance induced by an HFD. Numerous experimental and clinical studies have provided evidence supporting the gut microbiota as a potential target for controlling insulin resistance (14, 15). Consistent with many other studies (16), significant changes in the gut microbiota occurred in mice with insulin resistance induced by an HFD. Especially, 30 species belonging to *Alistipes* were significantly increased in HFD mice, such as *Alistipes finegoldii*, *Alistipes ihumii*, *Alistipes indistinctus*, *Alistipes obesi*, and *Alistipes timonensis*. Studies have shown that an animal-based diet increases the abundance of *Alistipes* in the gut microbiome (17). However, a recent study revealed that *A. indistinctus* improves insulin resistance in diet-induced obesity by affecting gut carbohydrate metabolism (14). Studies have shown that *Alistipes dysbiosis* can be either beneficial or harmful. For example, several diseases have been reported to be associated with an increased abundance of *Alistipes* in the gut microbiome, including irritable bowel syndrome, depression, and colorectal cancer (18–21). In contrast, other studies have shown that *Alistipes* may have protective effects against some diseases, including liver cirrhosis, colitis, and cardiovascular disease (22–24). Nevertheless, these findings indicate the important role of *Alistipes* in human health and disease, including insulin resistance, making them a potential target for disease diagnosis and treatment.

The abundances of four *Desulfovibrio* species and *C. Amulumruptor* were markedly lower in the HFD group compared to that in the ND group. *Desulfovibrio* has been linked to psychiatric disorder-associated gut microbiota and is significantly associated with obesity and type 2 diabetes (25). However, our results are inconsistent with these associations. Diverse phenotypes and functions can arise from different species or even different strains of the same species, such as *Faecalibacterium*, *Prevotella copri*, and *Eubacterium rectale*, as well as *Alistipes* mentioned earlier (26–28). Hence, understanding the genome variations that differentiate microbial strains is crucial. *C. Amulumruptor caecigallinarius*, a representative species of the *C. Amulumruptor* genus, was reconstructed from the chicken cecal microbiota and identified as a potential carbohydrate-digesting bacterium. However, the effects of *C. Amulumruptor* on the host have not been reported.

Further analysis found that signaling pathways of sphingolipid metabolism and propanoate metabolism, as well as the AGE-RAGE signaling pathway in diabetic complications, were significantly increased in HFD mice. Furthermore, these three metabolic functions exhibited a significant positive correlation with *Alistipes*, suggesting that *Alistipes* may contribute to insulin resistance by influencing host sphingolipid metabolism and pyruvate metabolism. Sphingolipids are vital signaling molecules in mammals, and abnormal levels of sphingolipids may result in weight gain, glucose intolerance, and insulin resistance (29, 30). The beneficial role of SCFAs on host metabolic health has been documented in numerous studies, encompassing obesity control, improved insulin sensitivity, and deceleration of diabetes progression (31–33). Nevertheless, some studies have reported contrasting effects of SCFAs. For instance, an increase in propionate was observed in insulin-resistant patients (14). Another study indicated that propionate intake could increase the secretion of glucagon and fatty acid-binding protein 4 (FABP4) in both mice and humans, potentially leading to insulin resistance (34). A recent study disclosed that fecal SCFA concentrations could not predict obesity (35). The conflicting nature of these results suggests that the relationship between SCFAs and host metabolic health remains unclear.

Aromatic amino acids (AAA) can be metabolized by the host and gut microbiota, where AAA metabolites regulate local and systemic immune, metabolic, and neuronal responses of the host. It has been reported to be related to various diseases, including gastrointestinal, liver, kidney, cardiovascular, and central nervous system diseases (36). AAA includes tryptophan, tyrosine, and phenylalanine. Through non-targeted metabolomics, we identified 21 metabolites derived from the microbiota or co-metabolized by the microbiota and host, which were associated with insulin resistance. These metabolites were predominantly enriched in tryptophan metabolism, tyrosine metabolism, and phenylalanine metabolism. Additionally, these metabolites were significantly correlated with the differential genera *Alistipes*, *C. Amulumruptor*, and *Desulfovibrio*, suggesting that these bacteria may influence host insulin resistance by producing/converting a range of AAA metabolites.

Various metabolites of tryptophan have different effects on the host. Qi et al. analyzed circulating tryptophan metabolite data from diverse racial backgrounds across five cohorts. They observed that circulating levels of tryptophan, four kynurenine pathway metabolites (kynurenine, kynurenate, xanthurenate, and quinolinate), and indole lactate were positively correlated with type 2 diabetes risk, whereas indolepropionate showed a negative association (37). Zhai et al. revealed that levels of *Ruminococcus gnavus-*derived metabolites tryptamine and phenethylamine play a causal role in the development of insulin resistance (38). In another study, it was found that increased indoleamine 2,3-dioxygenase activity altered the balance of tryptophan metabolism and the composition of the gut microbiota, thereby promoting obesity and diabetes (39). In animal and human experiments, researchers found that total parenteral nutrition reduces the level of tryptophan metabolites by changing the composition of the gut microbiota, thereby inhibiting the indole/Aryl hydrocarbon receptor (AhR) signaling pathway and the production of Glucagon-like peptide 1 (GLP-1), thus leading to glucose metabolism disorders (40). These studies highlight the crucial relationship between tryptophan metabolism in the gut microbiota and host metabolism. Our findings revealed that the levels of tryptophan, 5-hydroxyindole-3-acetic acid, and 4-(2-aminophenyl)-2,4-dioxobutanoic acid in the fecal metabolome of HFD mice were significantly lower than those of ND mice, suggesting a potential benefit in improving insulin resistance.

In the phenylalanine metabolism pathway, we observed enrichment of 3-coumaric acid, 2-phenylacetamide, and enol-phenylpyruvate in HFD mice, while ND mice exhibited higher levels of ortho-hydroxyphenylacetic acid and phenylacetaldehyde. In addition, the levels of dl-Dopa, tyramine, and indole-5,6-quinone from the pathway of tyrosine metabolism in ND mice were also higher than those in HFD mice. Currently, there is limited research on the relationship between these metabolites and host metabolism. 3-Coumaric acid, also known as hydroxycinnamic acids (HCA), has demonstrated the ability to regulate the gut microbiome and alleviate conditions such as intestinal ischemia–reperfusion injury and colorectal cancer in animal models. However, it is noteworthy that excessive HCA may have the potential to promote oxidation and cancer risk (41). Studies have demonstrated that 3-hydroxyphenylacetic acid improves spermatogenic dysfunction in aged mice, and 4-hydroxyphenylacetic acid mitigates obesity-driven hepatic steatosis in mice. These findings suggest a potential for ortho-hydroxyphenylacetic acid to also contribute to the improvement of host metabolism (42, 43).

In summary, our study contributes to previous reports regarding the involvement of gut microbiota and their metabolites in insulin resistance by integrating metagenomics and untargeted metabolomics. We identified potential gut microbiota and metabolite biomarkers, including new microbes and metabolites that have not been reported in previous insulin resistance-related studies. It is important to note that the identified microbes and metabolites associated with insulin resistance may be an effect of HFD, of which insulin resistance is only a common consequence. Future studies should consider including insulin-sensitive HFD mice to account for this factor, and employing a larger number of animals per group would enhance the robustness of the findings. Additionally, the identified microbes and metabolites associated with insulin resistance need to be further validated by experiments and further explore the mechanism of their influence on host insulin resistance. The use of germ-free animals and models will help to understand the role of these bacteria in disease and health and their interaction with host metabolism. Furthermore, attention should be given to the functional differences among different species and even strains.

## MATERIALS AND METHODS

### Animals and ethics approval

Approval for all animal experiments (No. 20191112091) was obtained from the Experimental Animal Welfare Committee of Zhejiang University of Technology, and these experiments adhered to the guidelines outlined in the “Experimental Animal Care and Use Guide” of Zhejiang University of Technology. We procured 12 four-week-old male C57BL/6J mice from Ziyuan Laboratory Animal Science and Technology (Hangzhou, Zhejiang, China). The mice were acclimated for 1 week in a specific pathogen-free environment with a temperature range of 22℃–25℃ and humidity was controlled with alternating 12-h light-dark cycles before any manipulation and were fed with autoclaved standard chow and water.

### Construction of an insulin resistance mouse model

Following 1 week of adaptive feeding, we randomly assigned the experimental mice into two groups. One group was fed an HFD (60% of calories derived from fat) and another group received an ND (10% of calories derived from fat). Details of the ingredients for both ND and HFD can be found in Table S4. The body weight and fasting blood glucose of mice were measured each week. Capillary blood glucose was obtained through a tail vein puncture using a handheld glucometer (Onetouch Verio Flex, Johnson & Johnson, USA). Prior to weekly blood glucose measurements, mice underwent a 12-h fasting period. Following a 15-week intervention, the body weight, serum fasting blood glucose levels, and HOMA-IR in HFD mice were significantly higher than those in ND mice (Fig. S1). After confirming the successful construction of the insulin resistance mouse model, the feces of all the experimental mice were collected and stored at −80°C until use.

### Metagenomics sequence and analysis

Total microbial DNA was extracted from the feces using a QIAamp Fast DNA Stool Mini Kit (Qiagen, Germany) according to the manufacturer’s instructions. The qualified DNA samples were sequenced on a Novaseq 6000 platform using a PE150 sequencing strategy (Illumina, USA). To ensure data accuracy and reliability, sequence reads were preprocessed by removing adapter sequences, low-quality sequences, and host genomic sequences. The clean reads were then subjected to an information analysis, including metagenome assembly, gene prediction, taxonomic classification, and functional annotation. Metagenome assembly was conducted using IDBA-UD (44). We used MetaGeneMark (45) to predict open reading frames (ORFs) from the assembled contigs with a length ≥500 bp. The ORFs with lengths less than 100 bp were filtered. A non-redundant gene catalog was constructed through pairwise comparison of all genes, utilizing CD-HIT (46) with criteria set at identity >95% and overlap >90%. Taxonomic assignments were determined using DIAMOND (diamond blastp --evalue ≤1e-5) (47) by aligning against the NCBI-NR database. KEGG annotations were made on the basis of DIAMOND (diamond blastp --evalue ≤1e-5) alignment against the KEGG protein database (48). Gene abundance was calculated by aligning the clean reads from each sample against to the gene catalog using Bowtie 2 (49). The abundance was normalized to Transcripts Per Million (TPM). The TPM normalization formula is $TPM=\frac{r_{k}}{L_{k}}\times \frac{1}{\sum_{i=1}^{n}\frac{r_{i}}{L_{i}}}\times 10^{6}$ , where *r* is the number of reads mapped to a gene sequence and *L* is the length of the gene sequence. The abundances of microbial taxa, KO, and KEGG pathway were calculated by adding the abundances of all the members falling within each category. Finally, information on genes, taxa, metabolic pathways, and potential functions in the gut microbiota, along with their respective abundances, was obtained.

### Non-targeted metabolomic profiling

Metabolites were extracted from fecal samples using the organic reagent precipitation protein method. At the same time, quality control samples are prepared by mixing equal amounts of prepared experimental samples. The metabolic profiling was performed by mass spectrum scanning in both positive and negative ion modes on LC–MS platforms. LC/MS raw data were converted into mzXML format and characterized using the ProteoWizard format through MSConvert software in Progenesis. XCMS software was used to perform the extraction of the peak area and quality control. Metabolite identification was conducted using MetaX software. The candidate metabolites were annotated using the HMDB (http://www.hmdb.ca/) and KEGG (https://www.genome.jp/kegg/) databases. MetaX software was used to quantify metabolites. Analysis of the experimental data was then conducted.

### Integrated metagenomic and untargeted metabolomic analyses

From the results of the metagenomic analysis, we identified significantly different bacterial genera and species, as well as KEGG metabolic pathways between the HFD and ND groups. Based on the results of untargeted metabolomics analysis, we obtained significantly different metabolites between the HFD and ND groups. Integrated analyses of metagenomics and untargeted metabolomics were performed on the MetOrigin website (https://metorigin.met-bioinformatics.cn/home/) (50). MetOrigin can also obtain sources of metabolites, metabolic pathways enriched by intergroups of different metabolites, and the interaction network of microbiome and metabolome. Multilevel regulatory relationships between significantly different gut microbes and metabolites were explored using Spearman analysis.

### Genomic analysis and functional prediction of candidate species

The protein sequences of the representative genome of 12 species belonging to *Alistipes*, *Desulfovibrio*, and *C. Amulumruptor* were downloaded from the NCBI RefSeq database. KO annotation and KEGG pathway analyses were performed using BlastKOALA or GhostKOALA tools on the KEGG website (https://www.genome.jp/kegg/) (51). We focused on the number of KOs annotated to eight metabolic pathways covering 21 significantly different metabolites in each genome. A higher number of KOs from a metabolic pathway in the genome implies a greater functional potential to participate in that metabolic pathway.

### Statistical analysis and visualization

The α-diversity of the compositions of bacterial species, including richness (the number of species) and the Shannon index, was calculated using the vegan R package. The comparison of α-diversity and microbial abundance between HFD and ND groups was performed using the Wilcoxon rank sum test, and a false discovery rate <0.05 was considered as the significance level. The vegan R-package was also used to perform PCoA. Adonis multivariate analysis of variance (Adonis) was performed to assess the difference in beta diversity between two groups. All significantly different bacterial species identified were visualized with Sankey diagrams using the networkD3 R-package. The heatmap was generated using the pheatmap R-package. Differential metabolites in positive and negative ion modes were screened based on three criteria: (i) fold change ≥2 or fold change ≤0.5, (ii) BH-corrected *P*-value (*q*-value) ≤0.05, and (iii) Variable Important for the Projection (VI) value obtained by multivariate statistical analysis of Partial Least Squares-Discriminant Analysis (PLS-DA) ≥1.

## Data Availability

Metagenomic and metabolomic data are available from the GitHub repository.

## SUPPLEMENTAL MATERIAL

Tables S1 to S4 spectrum.04094-23-s0001.xlsx Tables presented in an Excel document, with each table on a separate sheet.

Fig. S1 and S2 and legends of supplemental tables spectrum.04094-23-s0002.docx Supplemental figures and table legends

ASM does not own the copyrights to Supplemental Material that may be linked to, or accessed through, an article. The authors have granted ASM a non-exclusive, world-wide license to publish the Supplemental Material files. Please contact the corresponding author directly for reuse.
