# Supplementary material for: Screening potential biomarkers associated with insulin resistance in high-fat diet-fed mice by integrating metagenomics and untargeted metabolomics
Source: Microbiol Spectr. 2024 Feb 27;12(4):e04094-23. doi: 10.1128/spectrum.04094-23 (PMC10986473; doi:10.1128/spectrum.04094-23)
Supplement: Fig. S1 and S2 and legends of supplemental tables — Supplemental figures and table legends [file spectrum.04094-23-s0002.docx]

**Supplementary information for**

**Screening potential** **biomarkers** **associated with** **insulin resistance in high-fat diet-fed mice by integrating metagenomics and untargeted metabolomics**

Yunyan Zhou^a*#^, Jiahui Tang^a*^, Wei Du^b^, Yong Zhang^a^, Bang-Ce Ye^a,b#^

^a^Institute of Engineering Biology and Health, Collaborative Innovation Center of Yangtze River Delta Region Green Pharmaceuticals, College of Pharmaceutical Sciences, Zhejiang University of Technology, Hangzhou 310014, China.

^b^Laboratory of Biosystem and Microanalysis, State Key Laboratory of Bioreactor Engineering, East China University of Science and Technology, Shanghai 200237, China.

Running title: Biomarkers associated with insulin resistance

^#^Address correspondence to: [zyy@z](mailto:664280148@qq.com)jut.edu.cn (Yunyan Zhou); [bcye@ecust.edu.cn](mailto:bcye@ecust.edu.cn) (Bang-Ce Ye).

^*^Yunyan Zhou and Jiahui Tang contributed equally. Author order was determined on the basis of seniority.

**Supplementary Figures**

**
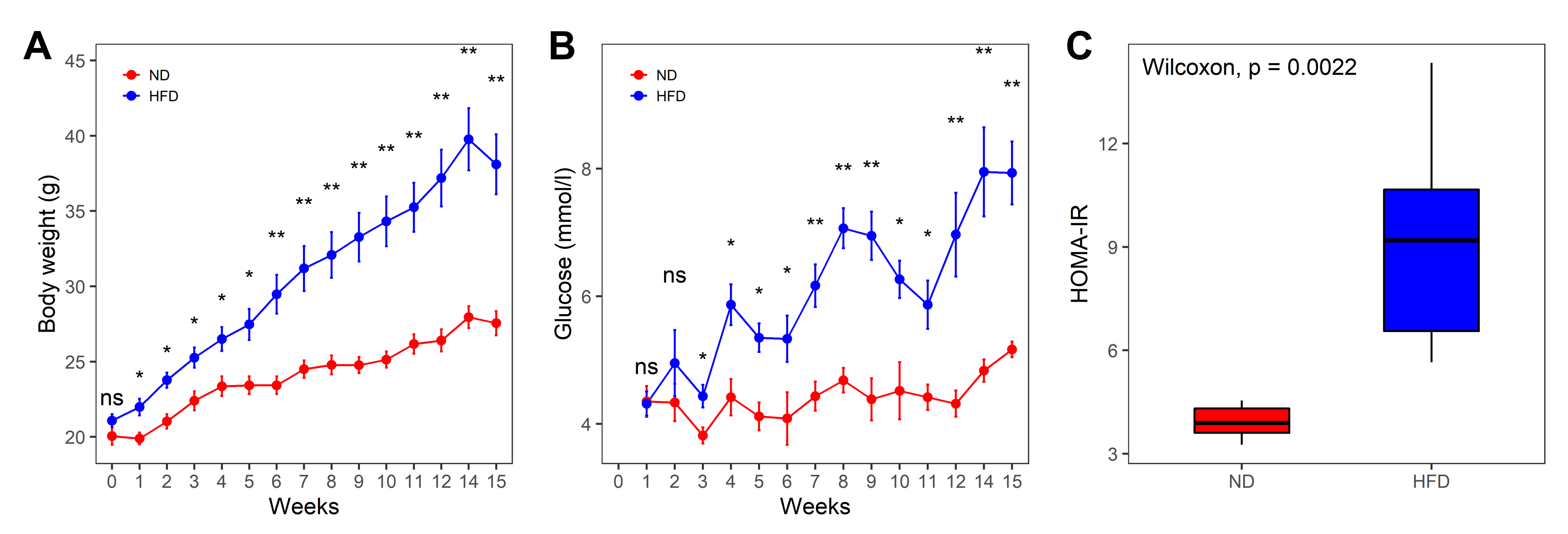
**

**Fig S1.** **Construction of insulin resistance mice model.** (A) The alterations in the body weight of mice fed high-fat (HFD) and normal (ND) diets during a 15-week intervention period. (B) The alterations in the serum fasting blood glucose levels of mice during a 15-week intervention period. (C) Comparison of homeostasis model assessment of insulin resistance (HOMA-IR) values between ND and HFD mice after the 15-week intervention. Six animals were used for each group. The comparisons were performed using the Wilcoxon rank sum test, and a P-value < 0.05 corrected for multiple tests (FDR) was set as the significance threshold.


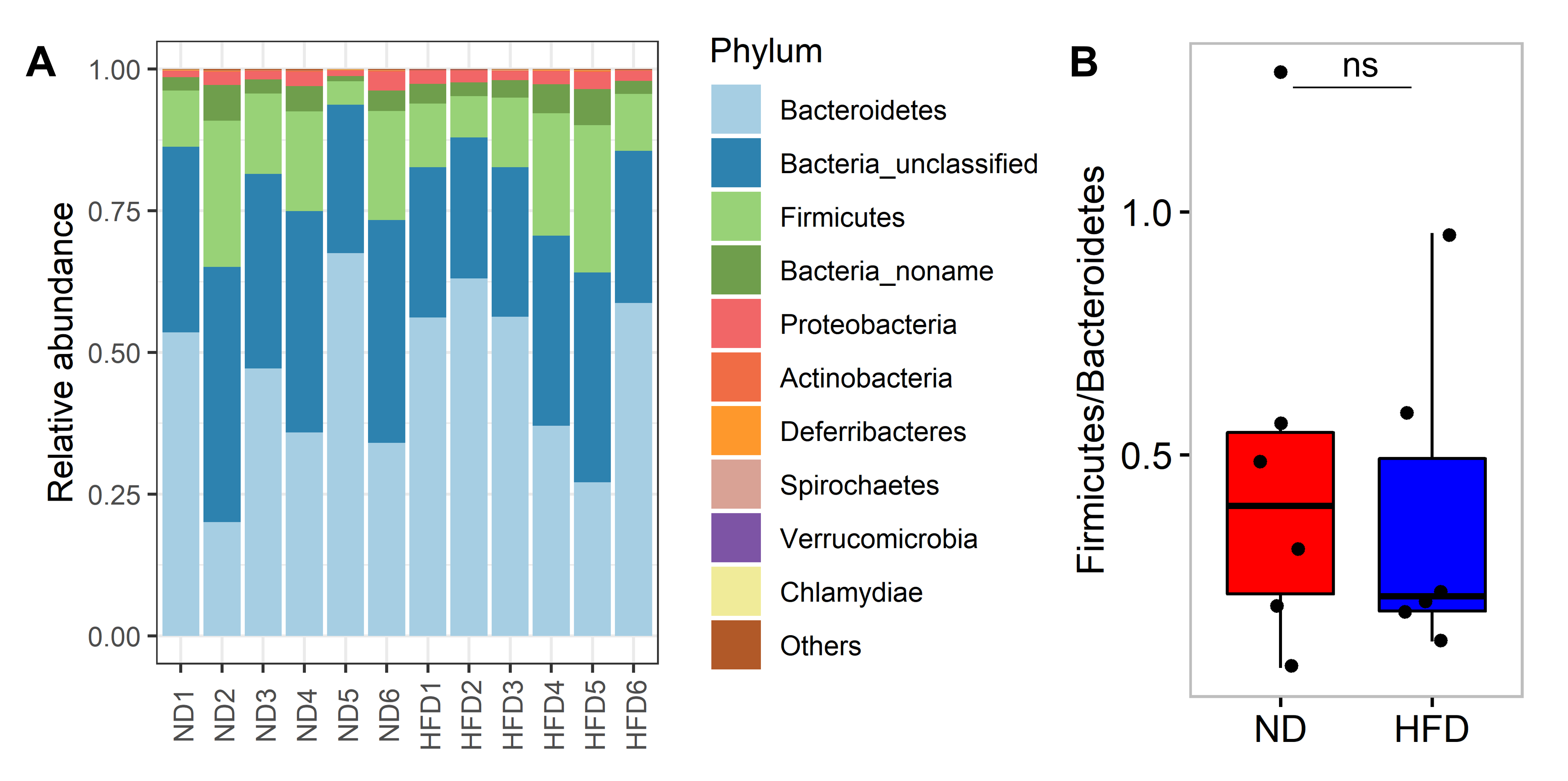


**FIG S2 The composition of gut microbiota communities at the phylum level.** (A) The relative abundance of the dominant phyla in each sample. (B) Comparison of the ratio of Firmicutes to Bacteroidetes between ND and HFD mice. The comparisons were performed using the Wilcoxon rank sum test, and a P-value < 0.05 corrected for multiple tests (FDR) was set as the significance threshold.

**Supplementary Tables**

Supplementary Tables are presented in an Excel document, with each table on a separate sheet.

**Table S1.** Bacterial species showing significant abundance differences between ND and HFD mice.

**Table S2.** Top 20 highly abundant species enriched in HFD and ND mice, respectively.

**Table S3.** Information on metabolic pathways enriched by 43 significantly different metabolites in ND and HFD mice.

**Table S4.** Ingredient composition of ND and HFD.
